# Unusual Root Canal Morphology of the Maxillary Second Molar: A Case Report

**DOI:** 10.1155/2013/138239

**Published:** 2013-03-14

**Authors:** Neslihan Şımşek, Ali Keleş, Elçin Tekın Bulut

**Affiliations:** Department of Endodontics, Faculty of Dentistry, Inonu University, 44280 Malatya, Turkey

## Abstract

*Introduction*. This clinical case report presents the successful endodontic treatment of a maxillary second molar that has a mandibular molar-like anatomy with no palatal root and with each of its roots containing two separate root canals. Cone-beam computed tomography (CBCT) was used to confirm this unusual anatomy. *Methods*. A 34-year-old male patient was referred to the Department of Endodontics at Inonu University's Faculty of Dentistry because of severe pain in his right maxillary second molar. Clinical and radiographic examinations identified unusual roots and root canals anatomy, and CBCT was planned in order to understand the nature of these variations. Cleaning and shaping procedures were performed using the crown down technique with Sybron Endo (Glendora, CA, USA) rotary instruments, and endodontic treatment was completed with gutta-percha cones and AH Plus resin sealers using the cold lateral compaction technique. *Conclusions*. The maxillary second molar exhibits aberrations and variations in terms of the numbers and configurations of its roots and root canals, and CBCT can be a useful imaging technique in endodontics.

## 1. Introduction 

It is known that thorough cleaning and shaping, in apical limits and dimensions, and obturation of all pulp spaces using an inert filling material are essential for root canal therapy. To perform successful endodontic treatment, it is important to have a clear understanding of the root canal system and its variations. The clinician who prepares the canal should expect, and be well equipped for, variations in the numbers of roots and root canal systems, in order to prevent undesirable consequences, including failure [1]. 

Most studies [2–6] of the morphology of the maxillary second molar have shown that the most frequently encountered type, known as standard morphology, has three roots: one mesiobuccal, one distobuccal, and one palatal. Each of these has a single canal. According to some studies [5–7], the presence of a second mesiobuccal canal is the most common variation found in the maxillary second molar. However, there are also case reports that present maxillary second molars with variations in the numbers of roots and root canals that they have. Benenati [8] presented a maxillary second molar with two palatal canals, and Deveaux [9] reported a maxillary second molar with two palatal roots. In another case report, Alani [10] described the endodontic treatment of four-rooted maxillary second molars that occur bilaterally. Fahid and Taintor [11] and Zmener and Peirano [12] also reported maxillary molars with three buccal roots, and Kottoor et al. [13] described a CBCT study that found a maxillary second molar with both five roots and five canals. 

This clinical case report presents a successful endodontic treatment of a maxillary second molar that has mandibular molar-like anatomy with no palatal root and with each of its roots containing two separate root canals. 

## 2. Case Report 

**Figure 1:**
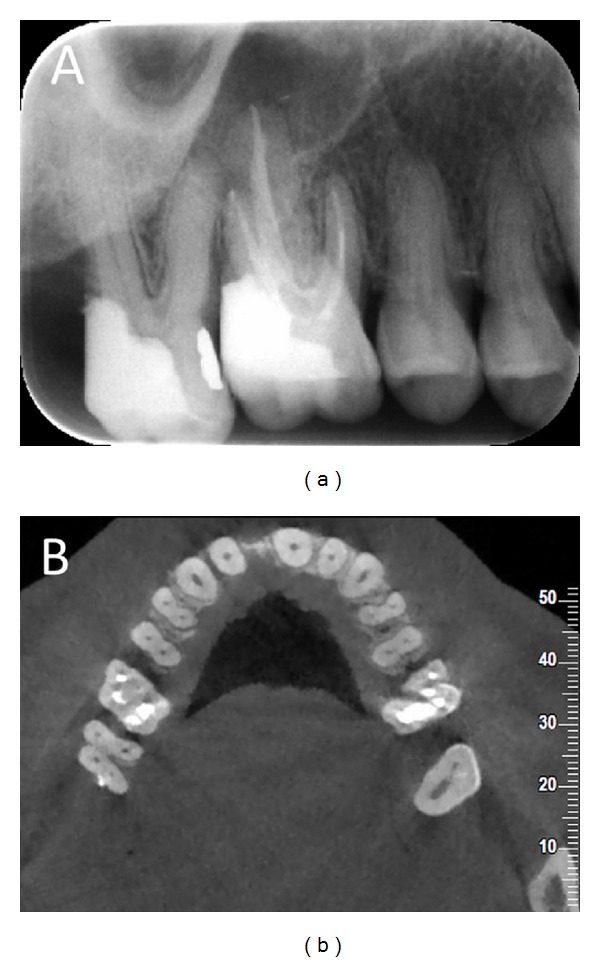
(a) Preoperative radiography showed that the tooth had two roots and no periapical radiolucency. (b) CBCT image revealed the absence of palatal root and two roots with two canals each.

A 34-year-old male patient was referred to the Department of Endodontics at Inonu University's Faculty of Dentistry because of severe pain in his right maxillary second molar. The patient's medical history was unremarkable. Clinical examination revealed that the tooth had a deep composite restoration and was not tender to palpation or percussion. The vitality tests were painful for both hot and cold stimulants, and electric pulp test was reduced. A preoperative radiograph showed no periapical radiolucency, and the periapical tissues were normal. Because radiographic examination revealed an unusual formation of the roots and root canals of the involved tooth, cone-beam computed tomography (CBCT-NewTom 5G, QR, Verona, Italy) was planned to acquire a better understanding of the nature of these variations. Informed consent was obtained from the patient. CBCT helped to verify the morphology of the roots and root canals. CBCT image demonstrated that the maxillary second molar tooth has two roots which were located mesially and distally, and each root has two canals (Figure 1). Based on the clinical examination and the patient's complaints, a diagnosis of irreversible pulpitis was established and endodontic treatment was planned.

**Figure 2:**
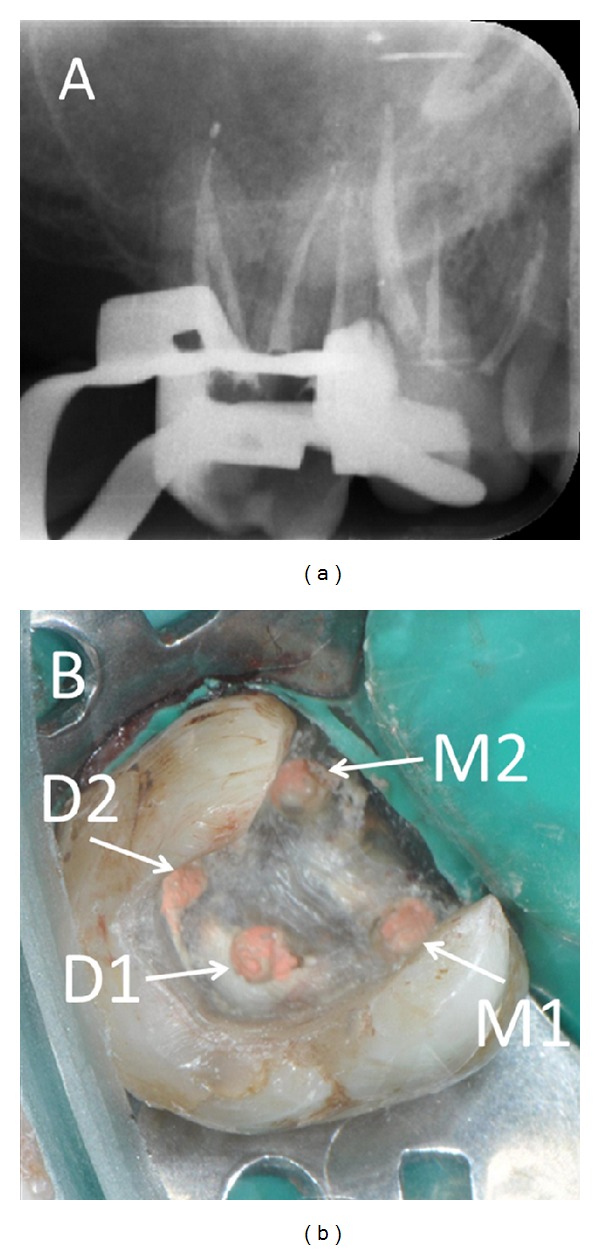
(a) Postoperative periapical radiograph taken immediately after completion of the root canal treatment. (b) The location of four root canal orifices after root canal filling.

**Figure 3:**
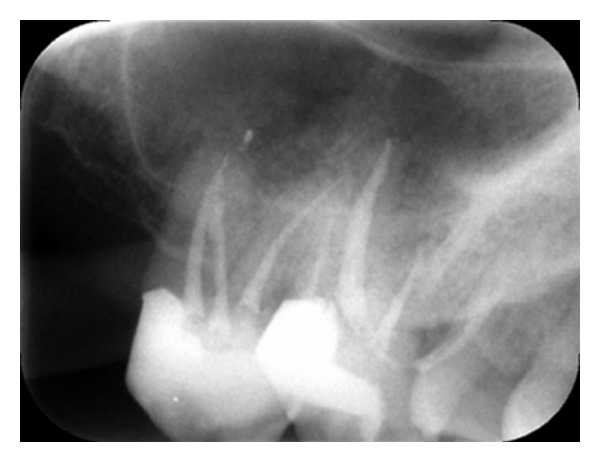
A 1-year follow-up periapical radiograph.

The tooth was anesthetized, and a rubber dam was put into place to isolate it. Preparation of the access cavity was also completed successfully. After a thorough inspection of the pulp chamber floor had confirmed the location of four root canal orifices, two of which were located mesially and two distally, these root canals were examined with no.10 K-type files (Mani Inc.). The working length was determined by Root ZX mini apex locator (J. Morita, Kyoto, Japan). To facilitate access to the root canals, coronal enlargement was prepared by using Gates Glidden burs (Mani, Inc., Tochigi, Japan) nos. 1, 2. Cleaning and shaping procedures were performed using twisted file (SybronEndo, Orange, CA) rotary instruments, according to the manufacturer's recommendations. The root canals were irrigated with 2.5% sodium hypochlorite between each of the instrumentations, and 17% EDTA (ethylenediamine tetraacetic acid) solution was used for the final irrigation. After the root canals had been dried, calcium hydroxide was applied as an intracanal medicament, a sterile cotton pellet was placed into the pulp chamber, and Cavit (ESPE, Seefeld, Germany) was used to seal the access cavity. A week later, the patient was asymptomatic. All root canals were obturated with gutta-percha cones and AH Plus (Dentsply De Trey GmbH, Konstanz, Germany) resin sealer, using the cold lateral compaction technique and a final periapical radiograph was taken to confirm the filling of the root canals (Figure 2). After one year, the clinical and radiographic followup revealed that the patient was asymptomatic and the periapical tissues and restored tooth were healthy (Figure 3).

## 3. Discussion

This case reports on a distinct formation of the roots and root canals of a maxillary second molar. Unlike some cases [9, 14, 15], which reported the presence of extra palatal roots in maxillary second molars, our case reveals the absence of a palatal root. In addition, in our report the roots of the maxillary second molar are positioned mesially and distally, and each has two root canals. In 1989, Libfeld and Rotstein [2] evaluated 1200 teeth in two groups: the first group included 1000 teeth and the second had 200. The study showed that 6% of the teeth in group one had two roots and 12% of those in group two had two roots with two canals. In addition, Peikoff et al. [4], who conducted a study of 520 completed endodontic treatments for maxillary second molars in 1996, described two separate roots, a buccal and a palatal, with one canal in each root as variant 4, with a percentage of 6.9%. A study by Lee et al. [7] also reported that, out of 205 maxillary second molars, 5.8% had two separate roots, with the variations of root canal anatomy frequently found in the buccal roots. Another recent study by Kim et al. [16] used CBCT to analyze the morphology of maxillary first and second molars in a Korean population and found the incidence of two separate roots in 821 maxillary second molars to be 9.8%. 

In this paper, the positions of the maxillary second molar's four root canal orifices were observed to be located mesiobuccally, mesiopalatally, distobuccally, and distopalatally, on the pulp chamber floor. Another recent study by Versiani et al. [17], who evaluated the root and root canal morphology of four-rooted maxillary second molars with micro-CT, described a new classification system, based on the configuration of the root canal orifices and their relationship to the pulp chamber floor, in maxillary second molars. Based on this classification system, our study categorized the involved maxillary second molar as type B (trapezoid shaped).

Conventional radiographic images force clinicians to visualize 3D structures based on 2D images, and the need to superimpose adjacent anatomic structures on each other for radiographs prevents clinicians from gathering clear, understandable images. CBCT imaging technology, however, with its 3D images eliminates the disadvantages of conventional radiographs [18]. In our case, even though periapical radiographs of the involved tooth revealed variations in root canal anatomy, they did not provide clear information about the nature of this variation. Using CBCT allowed us to acquire a better understanding of the variation and, therefore, to perform successful endodontic treatment. 

## 4. Conclusions 

The maxillary second molar exhibits aberrations and variations in terms of the numbers and configurations of its roots and root canals. Also, the complexity of the root canal system, especially in multirooted teeth, increases with the presence of such variations. CBCT can be a useful imaging technique in endodontics for providing a clear understanding of root canal morphology, especially when there are variations. 

## References

[1] Vertucci FJ (2005). Root canal morphology and its relationship to endodontic procedures. *Endodontic Topics*.

[2] Libfeld H, Rotstein I (1989). Incidence of four-rooted maxillary second molars: literature review and radiographic survey of 1,200 teeth. *Journal of Endodontics*.

[3] Neelakantan P, Subbarao C, Ahuja R, Subbarao CV, Gutmann JL (2010). Cone-beam computed tomography study of root and canal morphology of maxillary first and second molars in an Indian population. *Journal of Endodontics*.

[4] Peikoff MD, Christie WH, Fogel HM (1996). The maxillary second molar: variations in the number of roots and canals. *International Endodontic Journal*.

[5] Pecora JD, Woelfel JB, Sousa-Neto MD (1992). Morphologic study of the maxillary molars—part II: internal anatomy. *Brazilian Dental Journal*.

[6] Alavi AM, Opasanon A, Ng YL, Gulabivala K (2002). Root and canal morphology of Thai maxillary molars. *International Endodontic Journal*.

[7] Lee JH, Kim KD, Lee JK (2011). Mesiobuccal root canal anatomy of Korean maxillary first and second molars by cone-beam computed tomography. *Oral Surgery, Oral Medicine, Oral Pathology, Oral Radiology and Endodontology*.

[8] Benenati FW (1985). Maxillary second molar with two palatal canals and a palatogingival groove. *Journal of Endodontics*.

[9] Deveaux E (1999). Maxillary second molar with two palatal roots. *Journal of Endodontics*.

[10] Alani AH (2003). Endodontic treatment of bilaterally occurring 4-rooted maxillary second molars: case report. *Journal of the Canadian Dental Association*.

[11] Fahid A, Taintor JF (1988). Maxillary second molar with three buccal roots. *Journal of Endodontics*.

[12] Zmener O, Peirano A (1998). Endodontic therapy in a maxillary second molar with three buccal roots. *Journal of Endodontics*.

[13] Kottoor J, Hemamalathi S, Sudha R, Velmurugan N (2010). Maxillary second molar with 5 roots and 5 canals evaluated using cone beam computerized tomography: a case report. *Oral Surgery, Oral Medicine, Oral Pathology, Oral Radiology and Endodontology*.

[14] Ulusoy OIA, Görgül G (2007). Endodontic treatment of a maxillary second molar with 2 palatal roots: a case report. *Oral Surgery, Oral Medicine, Oral Pathology, Oral Radiology and Endodontology*.

[15] Shin SJ, Park JW, Lee JK, Hwang SW (2007). Unusual root canal anatomy in maxillary second molars: two case reports. *Oral Surgery, Oral Medicine, Oral Pathology, Oral Radiology and Endodontology*.

[16] Kim Y, Lee SJ, Woo J (2012). Morphology of maxillary first and second molars analyzed by cone-beam computed tomography in a korean population: variations in the number of roots and canals and the incidence of fusion. *Journal of Endodontics*.

[17] Versiani MA, Pecora JD, Sousa-Neto MD (2012). Root and root canal morphology of four-rooted maxillary second molars: a micro-computed tomography study. *Journal of Endodontics*.

[18] Cotton TP, Geisler TM, Hoden DT, Schwartz SA, Schindler WG (2007). Endodontic applications of cone-beam volumetric tomography. *Journal of Endodontics*.

